# Seipin deficiency increases chromocenter fragmentation and disrupts acrosome formation leading to male infertility

**DOI:** 10.1038/cddis.2015.188

**Published:** 2015-07-16

**Authors:** A E El Zowalaty, C Baumann, R Li, W Chen, R De La Fuente, X Ye

**Affiliations:** 1 Department of Physiology and Pharmacology, College of Veterinary Medicine, University of Georgia, Athens, GA, USA; 2 Interdisciplinary Toxicology Program, University of Georgia, Athens, GA, USA; 3 Department of Physiology, Georgia Regents University, Augusta, GA, USA

## Abstract

The Berardinelli–Seip congenital lipodystrophy type 2 (*Bscl2*, seipin) gene is involved in adipogenesis. *Bscl2*^−/−^ males were infertile but had normal mating behavior. Both *Bscl2*^−/−^ cauda epididymis sperm count and sperm motility were ~20 × less than control. *Bscl2*^−/−^ seminiferous tubules had relatively normal presence of spermatogonia and spermatocytes but had reduced spermatids and sperm. Spatiotemporal expression analyses in *Bscl2*^+/+^ testes demonstrated prominent *Bscl2* transcriptional activity in spermatocytes with a plateau reached around postnatal day 28. Seipin protein localization was most abundant in postmeiotic spermatids, suggesting translational repression of *Bscl2* mRNA in spermatocytes. *In situ* end-labeling plus detected increased spermatid apoptosis in *Bscl2*^−/−^ testis and annexin V detected increased percentage of positive *Bscl2*^−/−^ round spermatids compared with control. Immunofluorescence of marker proteins synaptonemal complex proteins 3 and 1 (SYCP3 and SYCP1), and H3K9me3 (histone H3 trimethylated at lysine 9) in germ cell spreads detected normal meiotic chromosome pairing and homologous chromosome synapsis in *Bscl2*^−/−^ spermatocytes, but significantly increased percentages of round spermatids with chromocenter fragmentation and late spermatids and sperm with chromatin vacuoles, indicating defective chromatin condensation in *Bscl2*^−/−^ spermatids. *Bscl2*^−/−^ late spermatids were disorganized within the seminiferous epithelium, despite normal appearance of Sertoli cells detected by vimentin immunofluorescence. Peanut agglutinin staining revealed various abnormalities of acrosomes in *Bscl2*^−/−^ late spermatids, including the absence, irregular-shaped, and fragmented acrosomes, indicating defective acrosome formation in *Bscl2*^−/−^ late spermatids, which may affect late spermatid orientation in the seminiferous epithelium. Mitotracker strongly stained the midpiece of control sperm but only very weakly labeled the midpiece of *Bscl2*^−/−^ sperm, indicating defective mitochondrial activity that most likely contributed to reduced *Bscl2*^−/−^ sperm motility. These data demonstrate novel roles of seipin in spermatid chromatin integrity, acrosome formation, and mitochondrial activity. Increased spermatid apoptosis, increased chromocenter fragmentation, defective chromatin condensation, abnormal acrosome formation, and defective mitochondrial activity contributed to decreased sperm production and defective sperm that resulted in *Bscl2*^−/−^ male infertility.

Berardinelli–Seip congenital lipodystrophy type 2 (*BSCL2*) gene encodes the protein seipin.^1^ Mutations of *BSCL2* are associated with generalized lipodystrophy characterized by a near-complete absence of adipose tissue and early-onset metabolic complications, such as insulin resistance and diabetes.^1, 2, 3^ Seipin is an integral endoplasmic reticulum (ER) membrane protein with two transmembrane domains, cytosolic N- and C-termini, and a central loop domain in the ER lumen.^4, 5^ Various adipocyte-associated functions of seipin have been proposed. It is found at ER lipid droplet junctions that are important for droplet morphology in yeast.^6^ In *Drosophila*, it has been shown to promote adipose tissue fat storage via physical interactions with SERCA (sarco/ER Ca^2+^-ATPase), which is an ER calcium pump solely responsible for transporting cytosolic calcium into the ER lumen.^7^ Seipin is also reported to regulate cyclic AMP/protein kinase A-mediated lipolysis that is essential for adipocyte maturation.^8^

Besides adipose tissue, seipin is also highly expressed in the testis.^1, 9, 10^ It was noticed that *Bscl2*^−/−^ males were infertile during previous study of seipin deficiency in adipose tissue loss and metabolic disorders.^8^ A recent study demonstrated that *BSCL2* mutations in a man and seipin deficiency in mice led to teratozoospermia and male infertility due to impaired testicular phospholipid homeostasis.^10^

*Bscl2*/seipin has been predominantly recognized for its role in adipogenesis^11, 12^ and phospholipid homeostasis.^10^ However, other potential roles, such as germ cell apoptosis and maintenance of DNA integrity, have been largely unexplored. Male germ cell apoptosis is a physiological process that maintains homeostasis in the seminiferous tubules during spermatogenesis. An early wave of male germ cell apoptosis occurs during prepubertal development and is thought to keep a proper balance between germ cells and supporting Sertoli cells.^13^ Germ cell apoptosis also occurs in seminiferous tubules of adult males^14^ and it is estimated that ~75% of all male germ cells produced are discarded through apoptosis as a mechanism to remove aberrant and excess germ cells.^15^ Interestingly, *Bscl2* was shown through transcription profiling of mouse embryonic stem (mES) cells to be transcriptionally activated on exposure to a broad spectrum of genotoxic compounds.^16^ This transcriptional activation of *Bscl2* was associated with inhibition of DNA replication and activation of the ataxia telangiectasia and Rad3-related protein (ATR) signaling pathway,^17^ suggesting that seipin might be involved in maintaining DNA integrity.

We aim to determine the role of seipin in male fertility using our *Bscl2*^−/−^ mouse model.^8^ Here we show that seipin deficiency disrupts spermatid DNA integrity, increases spermatid apoptosis, and interferes with acrosome formation.

## Results

### Bscl2^−/−^ male infertility due to reduced sperm production and impaired sperm motility

**Figure 1:**
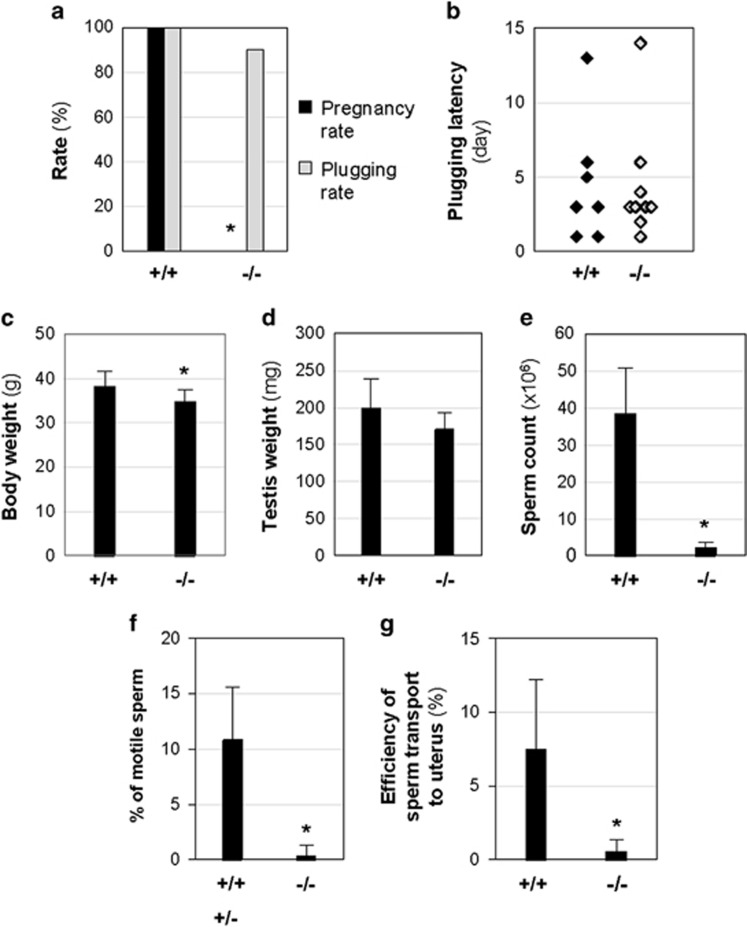
Male fertility test and sperm analyses. +/+, *Bscl2*^+/+^; +/−, *Bscl2*^+/−^; −/−, *Bscl2*^−/−^. (**a**) Pregnancy rate and plugging rate from 2 months of fertility test. (**b**) Plugging latency. (**c**) Body weight. (**d**) Testis weight. (**e**) Sperm count from cauda epididymis. (**a**–**e**): *N*=7 (+/+) and 10 (−/−). (**f**) Percentage of motile sperm. *N*=6 (+/+ and +/−) and 9 (−/−). (**g**) Efficiency of sperm transport to the uterus. *N*=5 (+/+) and 7 (−/−). Error bar, S.D.; *P<0.05

Two months of mating study revealed that all *Bscl2*^+/+^ females (*N*=7) mated with *Bscl2*^+/+^ males (100%) were pregnant. The average number of litters was 2.3±0.48 (*N*=7) and the average litter size was 8.8±3.0 (*N*=16 litters). However, none of the *Bscl2*^+/+^ females (*N*=10) mated with *Bscl2*^−/−^ males (*N*=10) became pregnant (0%), demonstrating that *Bscl2*^−/−^ males were infertile (Figure 1a). These *Bscl2*^−/−^ males had normal mating activities indicated by comparable plugging rate and plugging latency with *Bscl2*^+/+^ males (Figures 1a and b).

At the end of the fertility test, there were 9.3% reduction of body weight and 14.3% reduction of testis weight in *Bscl2*^−/−^ males (Figures 1c and d). There was no significant difference in the relative testis weight (Supplementary Figure S1A). However, *Bscl2*^−/−^ cauda epididymis sperm count was only ~5.4% of that from *Bscl2*^+/+^ males (Figure 1e). There was 15.8% reduction of absolute epididymis weight in *Bscl2*^−/−^ males (Supplementary Figure S1B) but comparable relative epididymis weight with *Bscl2*^+/+^ males (Supplementary Figure S1C). Interestingly, significantly increased absolute and relative weights of seminal vesicles and attached coagulating glands were observed in *Bscl2*^−/−^ males (Supplementary Figures S1D and E).

Sperm motility could contribute to male infertility and was analyzed in another set of males. The average sperm count in *Bscl2*^−/−^ group was ~3.3% of the control group (Supplementary Figure S1F). The average percentage of motile sperm in the same sperm preparations was ~0.4% from *Bscl2*^−/−^ males and ~10.8% from control males (Figure 1f). Sperm motility is essential for sperm transport in female reproductive tract.^18^ The efficiency of sperm transport was expressed as the percentage of the uterine sperm count over the cauda epididymis sperm count of the mated male, which was ~7.5% and ~0.5% in *Bscl2*^+/+^ and *Bscl2*^−/−^ males, respectively (Figure 1g, Supplementary Figures S1G and H). These data demonstrated reduced sperm count and defective sperm motility in *Bscl2*^−/−^ males.

### Histology showing defective spermatids and spermatozoa in Bscl2^−/−^ males

**Figure 2:**
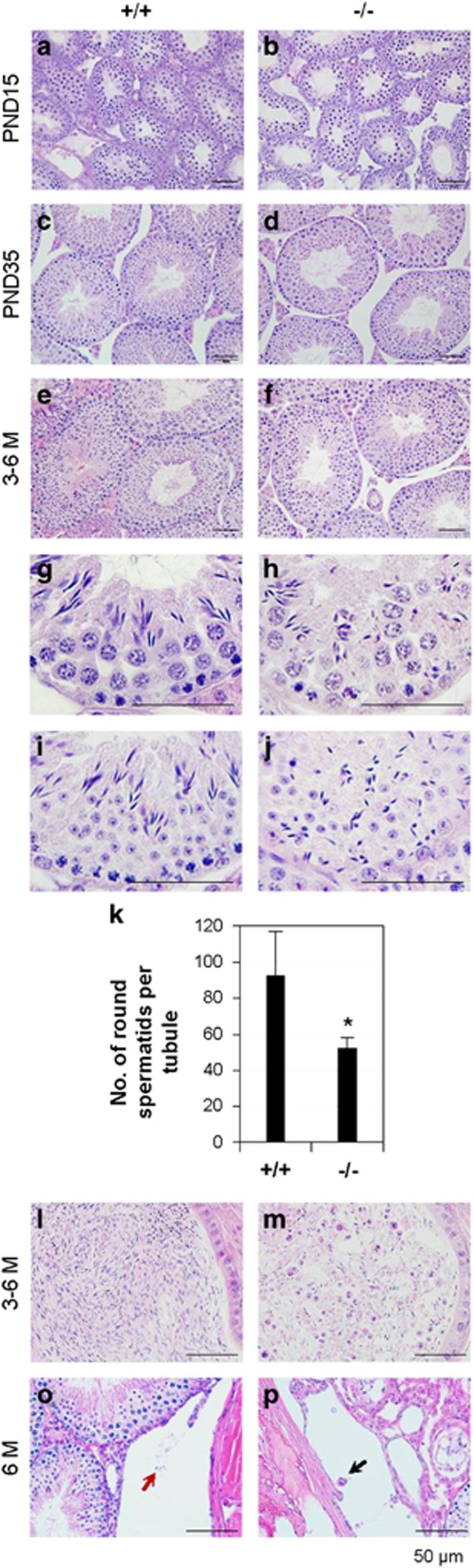
Histology of testis, cauda epididymis, and rete testis. +/+, *Bscl2*^+/+^; −/−, *Bscl2*^−/−^. Testes from three to six mice at each time point per genotype were analyzed. Representative images were shown. (**a**) PND15 *Bscl2*^+/+^ testis. (**b**) PND15 *Bscl2*^−/−^ testis. (**c**) PND35 *Bscl2*^+/+^ testis. (**d**) PND35 *Bscl2*^−/−^ testis. Data in **e**–**m** were from 3- to 6-month-old (3–6 M) males. (**e**) *Bscl2*^+/+^ testis. (**f**) *Bscl2*^−/−^ testis. (**g**–**j**) Enlarged views of *Bscl2*^+/+^ (**g** and **i**) and *Bscl2*^−/−^ (**h** and **j**) seminiferous epithelia to show disorientated distribution of spermatids in the *Bscl2*^−/−^ seminiferous epithelia. (**k**) Number of round spermatids per seminiferous tubule. *N*=5–6; error bar, S.D.; *P<0.05. (**l**) *Bscl2*^+/+^ cauda epididymis. (**m**) *Bscl2*^−/−^ cauda epididymis. (**o**) *Bscl2*^+/+^ rete testis. (**p**) *Bscl2*^−/−^ rete testis. **o** and **p**, arrows indicating germ cells. Scale bar, 50 *μ*m

Testis histology showed that the seminiferous epithelial thickness of the majority seminiferous tubules in *Bscl2*^−/−^ testes from postnatal day 15 (PND15) to adult was comparable to their age-matched *Bscl2*^+/+^ control (Figures 2a–f). Spermatogonia and spermatocytes appeared comparable between *Bscl2*^−/−^ and *Bscl2*^+/+^ seminiferous tubules. However, the spermatids, especially late-stage spermatids in the *Bscl2*^−/−^ seminiferous tubules appeared remarkably reduced (Figures 2e and f). The elongated *Bscl2*^−/−^ spermatids were often disorganized and were present throughout the seminiferous epithelium (Figures 2g–j). The disorganized spermatids prevented accurate staging of spermatogenesis in the *Bscl2*^−/−^ testes, except for stages IX to XI, which lack round spermatids. There was no difference in the average number of cross-sectioned seminiferous tubules per field ( × 20) (Supplementary Figure S1I) or the average number of stages IX to XI seminiferous tubules (Supplementary Figure S1J) between *Bscl2*^+/+^ and *Bscl2*^−/−^ testes. The average number of round spermatids per seminiferous tubule was significantly reduced in *Bscl2*^−/−^ testes (Figure 2k). The cauda epididymis sperm density from *Bscl2*^−/−^ males was dramatically decreased (Figures 2l and m) and there were many round apoptotic cell bodies in *Bscl2*^−/−^ cauda epididymis (Figure 2m) but not *Bscl2*^+/+^ cauda epididymis (Figure 2l). Rete testis histology showed scattered germ cells that were similar as those in the cauda epididymis (Figures 2o and p).

### Spatiotemporal expression of Bscl2/seipin in Bscl2^+/+^ testis

**Figure 3:**
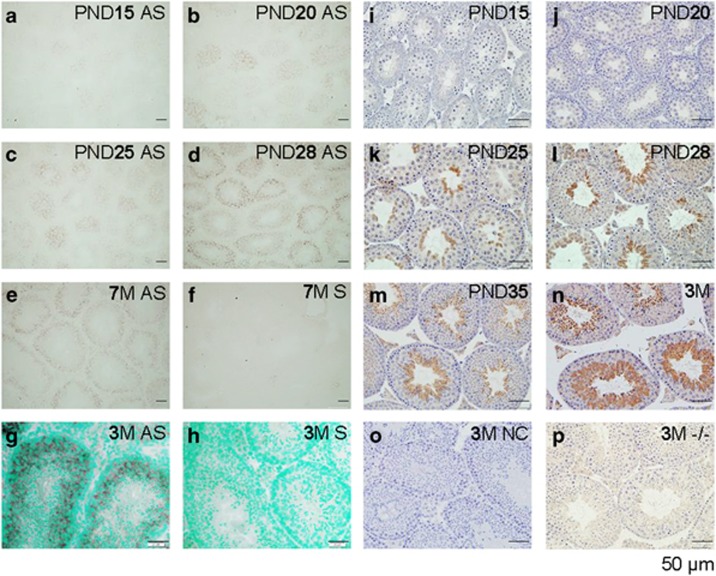
Spatiotemporal localization of *Bscl2* mRNA by *in situ* hybridization (**a**–**h**) and seipin protein by immunohistochemistry (**i**–**p**) in the testis. All sections were from *Bscl2*^+/+^ testes except **p**, which was from a *Bscl2*^−/−^ testis. Testes from at least three mice at each time point were analyzed. Representative images were shown. (**a**) PND15, antisense (AS) probe. (**b**) PND20, antisense probe. (**c**) PND25, antisense probe. (**d**) PND28, antisense probe. (**e**) 7-Month-old (7M), antisense probe. (**f**) 7M, sense probe, negative control. (**g**) 3M, antisense probe. (**h**) 3M, sense probe. (**g** and **h**) Counterstained with methyl green. (**i**–**p**) All sections were incubated with primary anti-seipin antibody, except **o**, which was incubated with normal rabbit IgG. (**i**) PND15. (**j**) PND20. (**k**) PND25. (**l**) PND28. (**m**) PND35. (**n**) 3M. (**o**) 3M, negative control (NC). (**p**) 3M, *Bscl2*^−/−^ (−/−), a second negative control. Scale bar, 50 *μ*m

Quantitative RT-PCR indicated >10-fold increase of *Bscl2* mRNA level in the 3-month-old *Bscl2*^+/+^ testes compared with PND15 *Bscl2*^+/+^ testis (Supplementary Figure S2A). *In-situ* hybridization revealed that *Bscl2* mRNA was barely detectable in most areas, except faint staining in some seminiferous tubules in the PND15 testis (Figure 3a) and gradually increased in PND20 (Figure 3b) and PND25 testes (Figure 3c). In PND28 testis, *Bscl2* mRNA was detected in all seminiferous tubules, although the levels of expression varied among different seminiferous tubules (Figure 3d). This *Bscl2* mRNA expression pattern remained throughout the 7-month-old testis, the oldest time point examined (Figure 3e). Within the seminiferous tubules, the highest expression levels were detected in the spermatocytes (Figure 3g). Based on a study about spermatogenic cells in prepubertal mouse testis,^19^ *Bscl2* mRNA was most likely expressed in both pachytene spermatocytes and secondary spermatocytes. *Bscl2* mRNA did not seem to be expressed in spermatogonia and Sertoli cells (Figure 3g). The signals in the interstitial spaces were insignificant (Figures 3d, e, and g). The negative control using a sense probe did not detect any positive signal (Figures 3f and h). Interestingly, *Bscl2* mRNA was highly and specifically detected in the epithelium of coagulating gland and seminal vesicle (Supplementary Figures S2B–F).

Immunohistochemistry analysis showed an age-dependent increase of seipin expression in the seminiferous tubules from PND15 to 3 months old (Figures 3i–n). On PND25, seipin started to be detected in the round spermatids and remarkably increased in the spermatids afterwards. IgG-negative control or *Bscl2*^−/−^ testis did not show specific staining (Figures 3o and p). Both histology (Figures 2a–f) and seipin expression suggested potential role of seipin in spermatid development or spermiogenesis.

### Increased ISEL^+^ staining in Bscl2^−/−^ testis

**Figure 4:**
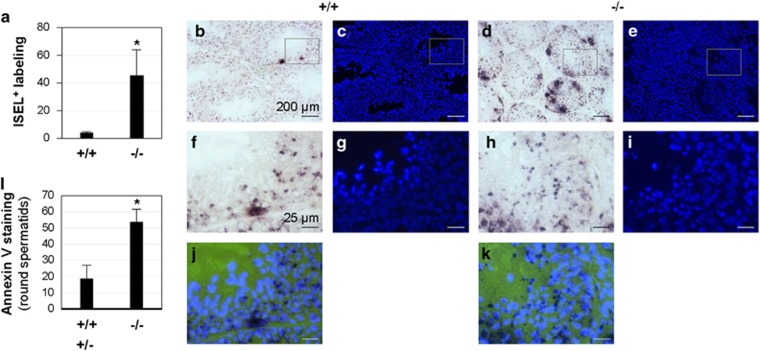
ISEL^+^ and annexin V staining of male germ cells. (**a**–**k**) ISEL^+^; 3- to 4-month-old *Bscl2*^+/+^ (+/+) and *Bscl2*^−/−^ (−/−) males; *N*=5. (**a**) Quantification of ISEL^+^ labeling using ImageJ as described in Materials and Methods. The original images used for quantification were in Supplementary Figure S4. (**b**) A representative ISEL^+^ testis section from a *Bscl2*^+/+^ male. (**c**) DAPI counterstaining of the section in **b**. (**d**) A representative ISEL^+^ testis section from a *Bscl2*^−/−^ male. (**e**) DAPI counterstaining of the section in **d**. Scale bars in **b–e**, 200 *μ*m. (**f**–**i**) Enlarged images of the rectangle areas in **b**–**e**, respectively. (**j**) Merged image of **f** and **g**. (**k**) Merged image of **h** and **i**. Scale bars in **f**–**k**, 25 *μ*m. Dark dots and clusters in **b**, **d**, **f**, **h**, **j**, and **k** were ISEL^+^ labeling. (**l**) Percentage of annexin V-positive round spermatids in 5-month-old control (*Bscl2*^+/+^ (+/+) and *Bscl2*^+/−^ (+/−), *N*=3) and *Bscl2*^−/−^ (−/−) (*N*=4) males. (**a** and **l**) *P<0.05; error bar, S.D.

Immunohistochemical analysis of cleaved caspase-3 did not show an obvious difference between *Bscl2*^+/+^ and *Bscl2*^−/−^ testes. A few cleaved caspase-3-positive germ cells were detected in both genotypes (Supplementary Figure S3). *In situ* end-labeling plus (ISEL^+^) detects DNA breaks.^20, 21^ Both *Bscl2*^+/+^ and *Bscl2*^−/−^ males had widespread ISEL^+^ labeling (Supplementary Figure S4). However, *Bscl2*^−/−^ testis sections (Supplementary Figure S4D) showed consistently more ISEL^+^ labeling than *Bscl2*^+/+^ testis sections (Supplementary Figure S4A). Quantification using ImageJ indicated a 10-fold increase of ISEL^+^ labeling intensity in *Bscl2*^−/−^ testis sections (Figure 4a), for which clusters of ISEL^+^ labeled cells were a main contributor (Figures 4b and d, and Supplementary Figure S4). Individual ISEL^+^ labeled cells not in the clusters were also detected in both *Bscl2*^+/+^ and *Bscl2*^−/−^ testis sections. These cells were located in the *Bscl2*^+/+^ seminiferous epithelium but rarely in the area close to the lumen where spermatids and sperm reside (Figures 4b, c, f, g, and j, and Supplementary Figures S4A–C). However, ISEL^+^ labeled cells were detected throughout *Bscl2*^−/−^ seminiferous epithelium, including the area where spermatids and sperm resided (Figures 4d, e, h, i, and k, and Supplementary Figure S4F), indicating increased DNA breaks and apoptosis of the *Bscl2*^−/−^ spermatids and sperm.

Annexin V staining detects cells in the intermediate stages of apoptosis. To support the association of increased DNA breaks in *Bscl2*^−/−^ late germ cells detected by ISEL^+^ with apoptosis, round spermatids were examined for annexin V staining in germ cell spreads. Results indicated significantly increased percentage of annexin V-positive *Bscl2*^−/−^ round spermatids (Figure 4l).

### Normal meiotic chromosome pairing and homologous chromosome synapsis in Bscl2^−/−^ spermatocytes

To determine the causes of DNA breaks in the late *Bscl2*^−/−^ germ cells, germ cell spreads from seminiferous tubules were analyzed. As *Bscl2* mRNA was mainly detected in the spermatocytes (Figure 3g), spermatocytes were examined. Pairing and subsequent synapsis of homologous chromosomes are essential developmental events necessary for proper meiotic recombination and chromosome segregation during metaphase I and II.^22^ The structural chromosome proteins synaptonemal complex proteins 3 and 1 (SYCP3 and SYCP1) are key components of axial (SYCP3) and lateral (SYCP1) elements of the synaptonemal complex^23, 24^ and were used as bona fide markers for prophase I of meiosis. SYCP3 was first detectable in leptotene-stage spermatocytes of both *Bscl2*^+/+^ and *Bscl2*^−/−^ preparations and progressively labeled the axial extension of the condensing chromosomes during zygotene and pachytene stages without indication for chromosome pairing errors (data not shown). This observation was also confirmed in synapsis of homologous chromosomes as indicated by co-localization of SYCP3 and SYCP1 in all autosomes at the pachytene stage of meiosis, irrespective of the genotypes (Supplementary Figure S5). In addition, the X and Y chromosomes were partially synapsed at the pseudoautosomal region in both *Bscl2*^+/+^ and *Bscl2*^−/−^ pachytene spreads (Supplementary Figure S5), indicating that *Bscl2*^−/−^ spermatocytes did not present obvious synaptic errors during prophase I.

### Chromocenter fragmentation in Bscl2^−/−^ round spermatids

**Figure 5:**
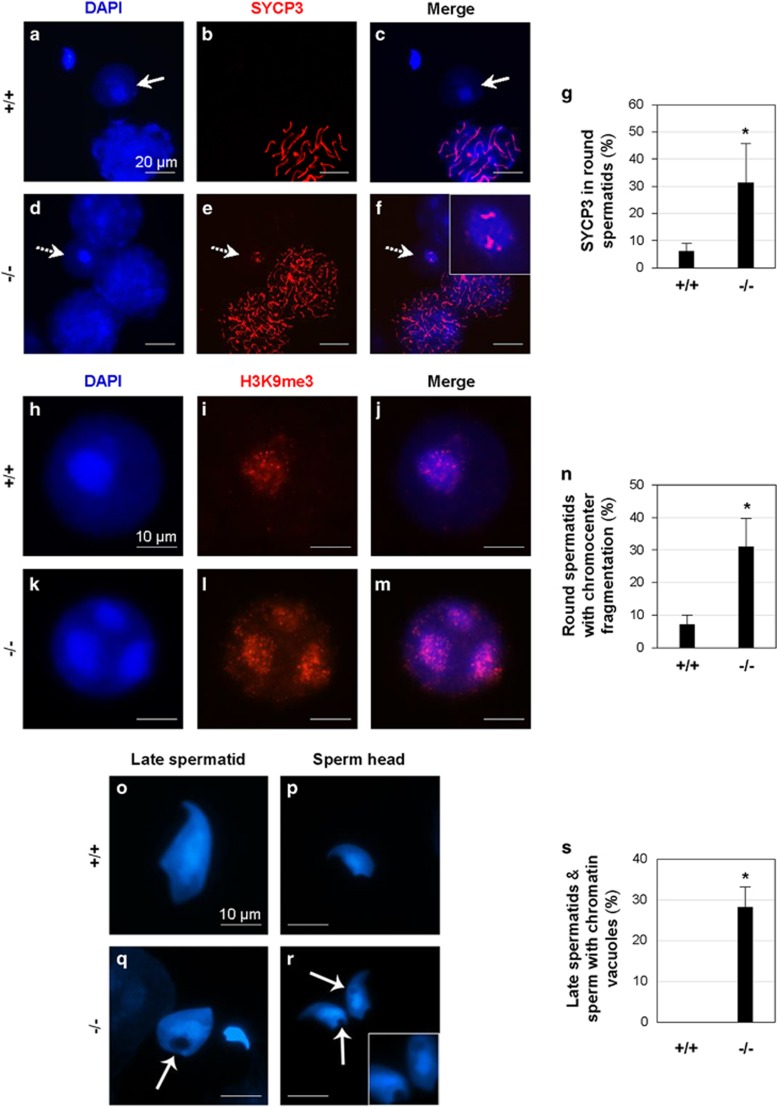
Analyses of round spermatids, late spermatids, and sperm in 4-month-old *Bscl2*^+/+^ (+/+) and *Bscl2*^−/−^ (−/−) germ cell spreads. (**a**–**c**) A representative image from *Bscl2*^+/+^ spreads; white arrows indicating a round spermatid. (**a**) DAPI. (**b**) SYCP3 staining the chromosomes in a pachytene spermatocyte in **a**. (**c**) Merged image of **a** and **b**. (**d**–**f**) A representative image from *Bscl2*^−/−^ spreads; white arrows with a broken line indicating a round spermatid. (**d**) DAPI. (**e**) SYCP3 staining the chromosomes in the zygotene spermatocytes and the round spermatid in **d**. (**f**) Merged image of **d** and **e**. Insert, enlarged view of the round spermatid with retained SYCP3 staining. (**g**) Percentage of round spermatids with retained SYCP3 staining. (**h**) Blue DAPI staining of a representative *Bscl2*^+/+^ round spermatid. (**i**) Red H3K9me3 staining of the round spermatid in **h**. (**j**) Merged image of **h** and **i**. (**k**) Blue DAPI staining of a representative *Bscl2*^−/−^ round spermatid. (**l**) Red H3K9me3 staining of the round spermatid in **k** showing chromocenter fragmentation. (**m**) Merged image of **k** and **l**. (**n**) Percentage of round spermatids with chromocenter fragmentation. (**o**–**r**) Representative DAPI staining of late spermatids and sperm. (**o**) A *Bscl2*^+/+^ late spermatid. (**p**) A *Bscl2*^+/+^ sperm head. (**q**) A *Bscl2*^−/−^ late spermatid and sperm head. (**r**) Two *Bscl2*^−/−^ sperm heads. Insert, enlarged view of the chromatin vacuoles. Arrowheads in **q** and **r**, chromatin vacuoles. Scale bar, 10 *μ*m. (**s**) Percentage of late spermatids and sperm with chromatin vacuoles. (**g**, **n**, and **s**) At least 100 randomly selected round spermatids from each sample were examined; *N*=3; error bars, S.D.; *P<0.05

As seipin is highly expressed in the postmeiotic spermatids (Figure 3), we analyzed global chromatin structure during spermiogenesis. SYCP3 is a marker of meiotic cells and is normally absent in the spermatids (Figures 5a–c).^25, 26^ However, there was an increased percentage of *Bscl2*^−/−^ round spermatids with retained SYCP3 expression (Figures 5d–g). Surface spreading of germ cell nuclei allows fine structural analyses of the sub-nuclear localization and distribution of chromatin domains. The chromocenter is a cluster of centromeres and pericentromeric heterochromatin.^27^ As a hallmark of spermiogenesis, centromeric heterochromatin domains of the autosomes coalesce to one or two central chromocenters with adjacent sex chromatin.^28^ In surface spreads obtained from *Bscl2*^−/−^ males, a significant proportion of round spermatids (31.0%, *P*=0.032) exhibited fragmentation of the chromocenter into three or more heterochromatic foci as demonstrated by labeling with a bona fide marker for centromeric heterochromatin, H3K9me3 (histone H3 trimethylated at lysine 9),^29^ whereas this phenotype was detectable in only 7.1% of *Bscl2*^+/+^ control (Figures 5h–n). These findings indicated abnormalities in the regulation of chromocenter formation in *Bscl2*^−/−^ males that may lead to defects in chromatin condensation and formation of proper spermatozoa and mature sperm.

### High incidence of chromatin vacuoles in Bscl2^−/−^ late spermatids and mature sperm

To address whether loss of seipin led to abnormal sperm chromatin condensation, morphological analyses were conducted using high-resolution microscopy of surface-spread germ cells. *Bscl2*^−/−^ late (elongating) spermatids and mature spermatozoa had a high incidence (28.2%, *P*<0.001) of chromatin vacuoles, which were not restricted to any particular nuclear domain, whereas such abnormalities were never observed in *Bscl2*^+/+^ germ cell preparations (Figures 5o–s).

Chromocenter fragmentation in round spermatids and chromatin vacuoles in late spermatids and mature sperms indicated potential problems in chromatin condensation, a process involving protamines 1 and 2 (PRM1/2) in mice.^27, 30, 31^ Although the levels of *Prm1* mRNA and *Prm2* mRNA, which are expressed in round spermatids,^32, 33^ were significantly reduced in 3-month-old *Bscl2*^−/−^ testes compared with *Bscl2*^+/+^ testes, the levels of PRM1 and PRM2 proteins, which are expressed in late spermatids and sperm, were comparable in the nuclei of individual *Bscl2*^+/+^ and *Bscl2*^−/−^ late spermatids and sperm (Supplementary Figure S6). Downregulation of *Prm1* and *Prm2* mRNAs in the *Bscl2*^−/−^ testis most likely resulted from the reduced number of round spermatids (Figure 2k and Supplementary Figures S6A and B). These data demonstrated that protamines most likely did not contribute to the chromatin defects in *Bscl2*^−/−^ late spermatids and sperm.

### Defective acrosome formation in Bscl2^−/−^ late spermatids

**Figure 6:**
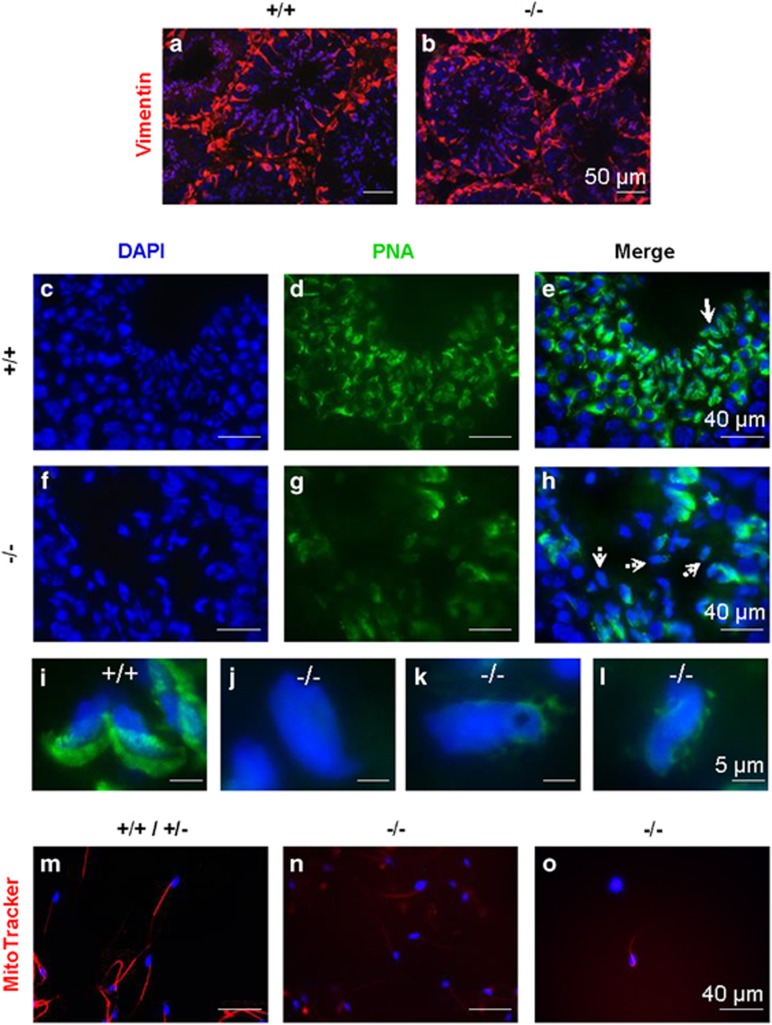
Detection of Sertoli cells, acrosomes, and active mitochondria. +/+, *Bscl2*^+/+^; −/−, *Bscl2*^−/−^. Sertoli cells were labeled by vimentin (red) immunofluorescence. Acrosomes were labeled with Alexa Fluor 488-conjugated PNA (green). Testes from 3- to 4-month-old mice (*N*=3–4) were used. (**a**) *Bscl2*^+/+^ testis vimentin labeling. (**b**) *Bscl2*^−/−^ testis vimentin labeling. (**c**) DAPI staining of *Bscl2*^+/+^ seminiferous epithelium. (**d**) PNA staining of the section in **c**. (**e**) Merged image of **c** and **d**. (**f**) DAPI staining of *Bscl2*^−/−^ seminiferous epithelium. (**g**) PNA staining of the section in **f**. (**h**) Merged image of **f** and **g**. (**i**) *Bscl2*^+/+^ spermatids indicated with solid white arrow in **e**. (**j**–**l**) *Bscl2*^−/−^ spermatids indicated with dashed white arrows in **h**. The spermatid in **k** had chromatin vacuole. Scale bar, 50 *μ*m in **a** and **b**, 40 *μ*m in **c**–**h**, and 5 *μ*m in **i**–**l**. (**m**–**o**) Red, MitoTracker staining of mitochondria at midpiece of sperm from 5-month-old cauda epididymis; blue, DAPI staining sperm head. (**m**) Representative control (*Bscl2*^+/+^ & *Bscl2*^+/−^) sperm with strong staining on midpiece. (**n)** Representative *Bscl2*^−/−^ sperm with faint staining. (**o**) A *Bscl2*^−/−^ sperm with strongest red stain in the group. Note, increased exposure in **n** and **o** to show *Bscl2*^−/−^ sperm midpiece

Besides chromocenter fragmentation in round spermatids and chromatin vacuoles in late spermatids and mature sperms, *Bscl2*^−/−^ late spermatids were disorganized and were present throughout seminiferous epithelium (Figures 2h and j, and Supplementary Figures S6I and R). Late spermatids are associated with Sertoli cells, which could be stained with vimentin. There was comparable vimentin staining between *Bscl2*^+/+^ and *Bscl2*^−/−^ testes (Figures 6a and b), indicating no obvious structural abnormality of the Sertoli cells that support sperm cell development before the spermatids are released to the lumen.

It was previously suggested that an acrosome may be related to the alignment of the spermatid head with the ectoplasmic specialization to influence the orientation and positioning of the late spermatids within the seminiferous epithelium.^34^ Peanut agglutinin (PNA) staining revealed crescent-shaped acrosomes in *Bscl2*^+/+^ late spermatids (Figures 6c–e and i). However, *Bscl2*^−/−^ spermatids had abnormal acrosomes (Figures 6f–h and j–l), such as no defined acrosome (Figure 6j), irregular-shaped acrosome (Figure 6k), and fragmented acrosome (Figure 6l). These data demonstrated that seipin deficiency also affected acrosome formation in late spermatids.

### Defective mitochondrion function in Bscl2^−/−^ sperm

Besides low sperm count (Figure 1e), the *Bscl2*^−/−^ sperm also had low sperm motility (Figure 1f). It has been demonstrated that there is direct and positive correlation between mitochondrial enzymatic activities and sperm motility.^35^ MitoTracker, which stains active mitochondria, was used to determine mitochondrial activity of sperm freshly isolated from the cauda epididymis. The majority of control sperm were motile and had strong staining at the midpiece (Figure 6m). However, the majority of *Bscl2*^−/−^ sperm were immotile and the MitoTracker staining was so weak that increased exposure time was required to visualize the flagellum (Figure 6n); even the strongest stained *Bscl2*^−/−^ sperm required increased exposure time (Figure 6o).

## Discussion

This study systemically analyzed the spatiotemporal expression of *Bscl2* mRNA and seipin protein in the mouse testis. Although both mRNA and protein levels gradually increased in the postnatal testes, with detectable levels by both *in situ* hybridization and immunohistochemistry after PND20, the mRNA levels seemed to reach a plateau ~PND28, while the protein levels were still increasing ~PND35. This was due to the main protein localization of seipin in spermatids. Round spermatids start to appear after PND18 and elongating spermatids start to appear ~PND35 and continue to develop till adulthood.^19, 36^ The temporal expression of seipin protein correlated with the onset of spermiogenesis and suggested its potential function in spermiogenesis. Indeed, in seipin-deficient testis, there was a reduction of round spermatids; escaping spermatids often lacked normal maturation features and showed signs of DNA damages; only a limited number of spermatids would go into the elongation phase, yet they would have aberrations such as chromatin vacuoles and abnormal acrosome formation; and the minimal number of sperm produced had diminished mitochondrial activity and motility.

Interestingly, *Bscl2* mRNA and seipin were not co-localized in the same male germ cells, with the mRNA mainly in the spermatocytes and the protein mainly in the spermatids, indicating translational repression in the spermatocytes. Translational repression in the male germ cells has also been reported for other genes in the testis. For example, mRNAs for transition proteins and protamines are synthesized in round spermatids but translated in elongated spermatids.^32, 33^

There were increased percentages of round spermatids with retained SYCP3 expression or chromocenter fragmentation in the *Bscl2*^−/−^ germ cell spreads. Although it is unknown about the significance of retained SYCP3 expression in the round spermatids, chromocenter fragmentation could lead to defects in chromatin condensation and the formation of proper spermatozoa and mature sperm. Chromocenter fragmentation has been reported in mice lacking the first bromodomain of testis-specific double bromodomain protein Brdt^37^ and mice deficient of TBP-like factor,^38^ both of which have impaired male fertility.^37, 38, 39^ Interestingly, the former was not associated with spermatid apoptosis^37^ but the later was associated with increased spermatid apoptosis.^39^

There were increased ISEL^+^ labeled cell clusters in *Bscl2*^−/−^ seminiferous epithelium as well as increased ISEL^+^ labeled individual spermatids at the center or close to the lumen of *Bscl2*^−/−^ seminiferous tubules. Male germ cell apoptosis is a normal process during spermatogenesis.^14^ It is most likely that chromocenter fragmentation contributed to the increased spermatid apoptosis in *Bscl2*^−/−^ testis. However, the causes for increased ISEL^+^ labeled cell clusters in the *Bscl2*^−/−^ seminiferous epithelium are unknown. Such clusters were also seen in the *Bscl2*^+/+^ seminiferous epithelium but at a much less frequency. As spermatids are connected by cytoplasmic bridges and *Bscl2*^−/−^ spermatids were often observed in abnormal locations near the periphery of seminiferous tubule, it is possible that the increased ISEL^+^ labeled cell clusters near the base of seminiferous epithelium was due to increased *Bscl2*^−/−^ spermatid apoptosis. In addition, as germ cells at different stages are supported by the same Sertoli cells, it is unknown whether the defects in the spermatids could affect earlier germ cells that share the same Sertoli cells. The increased germ cell death in the *Bscl2*^−/−^ testis was obviously unassociated with caspase-3 pathway. This scenario had been reported in RARalpha^−/−^ testes as well,^40^ which also had disorganized late spermatids and increased spermatid apoptosis.

Chromatin vacuoles were only observed in late spermatids and spermatozoa from the *Bscl2*^−/−^ testis. As the localizations of the chromatin vacuoles could be found in different areas, it is possible that these vacuoles are formed by defects in large-scale chromatin condensation during spermatid elongation and thus had much less density on DAPI (4',6-diamino-2-phenylindole) staining. It is possible that defects in chromatin condensation might be associated with chromocenter fragmentation in round spermatids. One study revealed that the small vacuoles in human sperm were abnormal nuclear concavities associated with noncondensed chromatin.^41^ Chromatin vacuoles are also observed in sperm deficient of chromodomain helicase DNA-binding protein 5.^27^

One defect in the *Bscl2*^−/−^ testis was the disorganized late spermatids. Although the role of acrosome in spermatid orientation has not been established, it was suggested decades ago that an acrosome may have a role in aligning the spermatid head with the ectoplasmic specialization to influence the orientation and positioning of the late spermatids.^34^ The *Bscl2*^−/−^ spermatids had various defects in their acrosomes and were often disorganized. Seipin was shown to be localized on the ER membrane.^4, 5^ ER is closely associated with the Golgi apparatus where the acrosome is derived from. It is possible that seipin is directly involved in acrosome formation in late spermatids.

A recent study on *Bscl2*^−/−^ testes from a different line of global seipin knockout mice and germ cell conditional knockout mice demonstrated that the defective sperm production was due to local deficiency of seipin in the germ cells.^10^ The percentages of motile sperm were much lower in our study, which was due to different sample preparations. Increased phosphatidic acid (PA) level was observed in the *Bscl2*^−/−^ testis.^10^ Phospholipases A PLA_1_ and PLA_2_ are involved in PA metabolism. PA-preferring PLA_1_ (PA-PLA_1_) is highly expressed in the testis. PA-PLA_1_-deficient sperm have a bent flagellum and severely impaired sperm motility.^42^ Sperm tail defects were also observed in the *Bscl2*^−/−^ sperm,^10^ which might be related to the function of PA-PLA_1_ in flagellum and sperm motility.^42^ In addition, lack of active mitochondria in the *Bscl2*^−/−^ sperm midpiece could be a main reason for defective sperm motility.

In addition to its critical role in adipogenesis,^11, 12^ *Bscl2*/seipin most likely has other uncharacterized roles. Its transcriptional upregulation in mES cells on exposure to genotoxins involving the ATR signaling pathway^16, 17^ would suggest its potential role in maintaining DNA integrity on genotoxic insults. Although testicular-derived seipin was suggested to be essential for male fertility by modulating testicular phospholipid homeostasis,^10^ we assume that defective lipid homeostasis in *Bscl2*^−/−^ male germ cells could contribute to a defective flagellum with low mitochondrial activity and thus impaired sperm motility, whereas impaired DNA integrity and chromatin condensation could contribute to both reduced sperm count and impaired sperm motility, because DNA damage can induce apoptosis and is negatively related with sperm motility.^43, 44^ Reduced sperm count, reduced sperm motility, and defective acrosome formation contribute to male infertility in the *Bscl2*^−/−^ mice.

## Materials and Methods

### Animals

*Bscl2*-deficient mice (*Bscl2*^−/−^) in C57BL/6 J background were derived from a colony at Georgia Regents University, which was originally derived from a colony at Baylor College of Medicine, with backcrosses to C57BL/6 J background for five generations.^8^ They were genotyped as previously described,^8^ using mouse tail genomic DNA and PCR with wild-type allele-specific forward primer 5′-GGACGTGATCGGGTGAGTATGAGAA-3′ and reverse primer 5′-GAGATAGGGTCTGGCTATGAA-3′, and the neo-specific primer 5′-CTATCGCCTTCTTGACGAGT-3′. All mice were maintained on PicoLab mouse diet 20 with soybean as a main protein source. They were housed in polypropylene cages with free access to food and water on a 12 h light/dark cycle (0600–1800 h) at 23±1 °C with 30–50% relative humidity at the College of Veterinary Medicine animal facility at the University of Georgia. The animals were killed by CO_2_ inhalation followed with cervical dislocation. All methods used in this study were approved by the University of Georgia Institutional Animal Care and Use Committee (IACUC) Committee and conform to National Institutes of Health guidelines and public law.

### Male mating behavior and fertility tests

*Bscl2*^+/+^ males (*N*=7) and *Bscl2*^−/−^ males (*N*=10) at 2–3 months old were each housed with one 2-month-old virgin *Bscl2*^+/+^ female for 2 months. Each female was checked for the presence of a vaginal plug every morning, to determine mating activity during the previous night. Mating activity was evaluated with two parameters, plugging latency (the duration between cohabitation and the detection of the first plug) and plugging rate (the percentage of males in each group that ever successfully mated with females during the 2 months of cohabitation). Fertility was assessed using the litter size at birth and the number of litters per female. At the end of the 2-month-cohabitation, the males were separated from the mating females and the females were observed for 3 more weeks for pregnancies that occurred during the last period of mating study. The average ages of males were ~20 weeks old for both *Bscl2*^+/+^ (20.4±3.8 weeks) and *Bscl2*^−/−^ (20.0±2.3 weeks) males. Two *Bscl2*^+/+^ males and three *Bscl2*^−/−^ males that finished mating and fertility tests before the rest males were killed for sperm counting only, the rest five *Bscl2*^+/+^ males (20.6±4.4 weeks old) and seven *Bscl2*^−/−^ males (19.9±0.6 weeks old) were mated with one virgin female (2–3 months old) each for determining sperm count in the uterus before being killed for sperm counting. Positive mating was determined by the presence of a vaginal plug in the morning and the uterine horns from the positively mated females were flushed with 1 × PBS for sperm counting from the uterus. Cauda epididymis sperm count was determined 1 week after mating and uterine sperm count was determined in the morning after mating. The efficiency of sperm transport was expressed as the percentage of the uterine sperm count over the cauda epididymis sperm count of the mated male.

### Sperm count and sperm motility

All males were housed individually for 1 week before sperm counting. Each cauda epididymis was dissected, finely minced in 1 ml 1 × PBS in a 1.5-ml microcentrifuge tube, and shaken at 37 °C/5% CO_2_ for 30 min. Sperm count was expressed as the total sperm number from both cauda epididymides. As sperm motility was not determined in the males from mating study, another set of males was used. Both *Bscl2*^+/+^ and *Bscl2*^+/-^ males were used in the control group, because they did not show any obvious difference in the phenotypes. Cauda epididymides from control males (*N*=6, 25.1±6.4 weeks old) and *Bscl2*^−/−^ males (*N*=9, 24.0±5.1 weeks old) were dissected and prepared for sperm counting as described above. Meanwhile, the number of motile sperm from each preparation was also determined. Sperm motility was expressed as the percentage of motile sperm. The sperm number in the uterine flushing from each of the females mated with a subset of males in the fertility test was also counted using a hemocytometer.

### Testis and rete testis histology and spermatogenesis staging

Testes from *Bscl2*^+/+^ and *Bscl2*^−/−^ males at PND15, PND35, and 3–6 months old, as well as testes together with rete testis and epididymis from 6-month-old males were fixed in Bouin's solution (Ricca Chemical Company, Arlington, TX, USA) for 24 h. They were then kept in 70% ethanol until embedded in paraffin for sectioning. Each testis was cut perpendicular to the long axis of the seminiferous tubules. Paraffin sections (5 *μ*m) were processed and stained with hematoxylin and eosin.^21^ *N*=3–6 for each time point and each genotype. To determine spermatogenesis stages, a representative × 20 testis histology image with only cross-sectioned round seminiferous tubules from each adult mouse (3–6 months old) was analyzed. As spermatids in the *Bscl2*^−/−^ seminiferous tubules were disorientated and prevented accurate staging, the following information was collected: the number of intact seminiferous tubules in each image, the number of seminiferous tubules without round spermatids (stage IX–XI), and the total number of round spermatids in all intact seminiferous tubules in each × 20 image. Histology of rete testes was obtained by sectioning through the testis and rete testis, and stained with hematoxylin and eosin as described above.

### Quantitative RT-PCR

Testes from wild-type C57BL/6J males at PND15 (*N*=5) and 3-month-old mice (*N*=3) were snap frozen on dry ice. Total RNA was extracted with Trizol (Life Technologies, Grand Island, NY, USA). Complementary DNA (cDNA) was transcribed using Superscript III reverse transcriptase (Life Technologies) with random primers as previously described.^21, 45, 46^ Quantitative PCR was performed in 384-well plates using SYBR-Green I intercalating dye on ABI 7900 (Applied Biosystems, Carlsbad, CA, USA). The mRNA expression levels of *Bscl2* were determined using gene-specific primers from different exons (Integrated DNA Technology, Coralville, IA, USA), m*Bscl2*e3F: 5′-GTGCACTTCCACTACAGGAC-3′ and m*Bscl2*e6R: 5′-CTCCAGTTGTTGGCACATAC-3′. *Gapdh* (glyceraldehyde-3-phosphate dehydrogenase) was used as a loading control and *Hprt1* (hypoxanthine phosphoribosyltransferase 1), another housekeeping gene, served as a second control as previously described.^47^ For quantification of *Prm1* and *Prm2*, total RNA was extracted from 3-month-old *Bscl2*^+/+^ and *Bscl2*^−/−^ males (*N*=5) with Trizol (Life Technologies) and cDNA was transcribed as described above. Quantitative PCR was performed using Ssoadvanced SYBR Green Supermix (Bio-Rad, Hercules, CA, USA) on CFX384 Touch Real-Time PCR Detection System (Bio-Rad). Gene-specific primers (Integrated DNA Technology) were designed as reported previously:^48^ m*Prm1*e2F: 5′-AGGTGTAAAAAATACTAGATGCACAGAATAG-3′, m*Prm1*e2R: 5′-TTCAAGATGTGGCGAGATGCT-3′ and m*Prm2*e2F: 5′-GAATAGTCACCTGCCCAAGCA-3′, m*Prm2*e2R: 5′-GCAGCTCAGGGCTCAGACA-3′. *Gapdh* and *Hprt1* were used as control as described above.

### *In situ* hybridization

*Bscl2* sense and antisense cRNA probes were synthesized as previously described,^49^ except that the template for cRNA probe synthesis was amplified from m*Bscl2* cDNA using primers m*Bscl2*e3F and m*Bscl2*e6R for PCR. The amplified *Bscl2* cDNA fragment was recovered from agarose gel and cloned into pGEM-T Easy Vector (Promega, Madison, WI, USA). The recombinant plasmid was amplified with T7 and SP6 primers to produce templates for labeling antisense and sense probes, respectively. Testes from wild-type males at PND15, PND20, PND28, PND35, 3 months old, and 7 months old were snap frozen on dry ice and kept at −80 °C. Frozen testis cross-sections (10 *μ*m) were cut and processed. Testis sections were mounted on (3-aminopropyl)triethoxy silane-coated slides, fixed in 4% paraformaldehyde in diethylpyrocarbonate (DEPC)-treated H_2_O for 1 h at room temperature. Sections were washed twice with 1 × PBS in DEPC H_2_O for 5 min, treated with 1% Triton X-100 for 20 min, then washed three times in 1 × PBS for 5 min each. Sections were prehybridized in 50% formaldehyde/5 × saline-sodium citrate (SSC) buffer at room temperature for 15 min and then hybridized with digoxigenin-labeled sense (as a negative control) or antisense riboprobes for 16–20 h. Sections were next washed in 5 × SSC, 50% formamide at 55 °C for 15 min; 2 × SSC, 50% formamide at 55 °C for 30 min; 0.2 × SSC, 50% formamide twice at 55 °C for 30 min each; and 0.2 × SSC at room temperature for 5 min, then washed in buffer B1 (1 M Tris-HCl pH7.5, 2.5 M NaCl in ddH_2_O) for 5 min at room temperature. Slides were incubated with 1% blocking agent (Boehringer, Mannheim, Germany) in buffer B1 at room temperature for 1 h. Slides were next incubated with 1 : 2000 Anti-Digoxigenin-AP, fab fragment (Roche Applied Science, Indianapolis, IN, USA) in 1% blocking reagent at 4 °C overnight. After being washed three times in buffer B1 for 5 min each, slides were washed in buffer B3 (1 M Tris-HCl pH 9.5, 0.5 M MgCl_2_, 2.5 M NaCl in ddH_2_O) for 5 min at room temperature. The hybridization was visualized with substrates nitroblue tetrazolium and 5-bromo-4-chloro-3-indolyl phosphate (Roche Applied Science) in buffer B3, and endogenous alkaline phosphatase activity was inhibited with 2 mM Levamisole (Sigma-Aldrich, St Louis, MO, USA) added to the substrate solution. Some sections were counterstained with methyl green as previously described.^49, 50^ Testes from at least three different mice were examined for each time point.

### Immunohistochemistry

Paraffin sections (5 *μ*m) were used to detect seipin expression in the testes from PND15, PND20, PND35, and 3-month-old wild-type mice using our customized rabbit polyclonal anti-seipin antibody (1 : 1000, 2.21 *μ*g/ml, Thermo Fisher Scientific, Waltham, MA, USA), which was raised against the C-terminal 17 amino acids of mouse seipin as previously described.^51^ Briefly, testis sections were rehydrated, subjected to antigen retrieval in 10 mM sodium citrate (pH 6) for 20 min in a microwave, and then washed in 1 × PBS and ddH_2_O for 5 min each. All steps were carried out at room temperature unless specified. Endogenous horseradish peroxidase (HRP) was blocked with 3% H_2_O_2_ in methanol for 10 min and then washed in ddH_2_O and 1 × PBS for 5 min, respectively. Nonspecific staining was blocked with 10% goat serum for 1 h. Sections were then incubated with rabbit anti-seipin overnight at 4 °C. Sections were washed in 1x PBS for 5 min, incubated with biotinylated goat anti-rabbit IgG (1 : 200, 7.5 *μ*g/ml, BA-1000, Vector Laboratories, Burlingame, CA, USA) in 1% BSA for 30 min. After washing three times with 1 × PBS for 5 min each, sections were incubated with HRP streptavidin (SA-5004, Vector Laboratories) for 30 min and then washed three times in 1 × PBS for 5 min each. After development with 3,3' diaminobenzidine tetrahydrochloride (0.03%–0.05% in 0.05 M Tris-HCl pH 7.6, 0.01%–0.03% H_2_O_2_) for 5–10 min, sections were counterstained with hematoxylin, dehydrated, cleared, and mounted. Cleaved caspase-3 was detected in testes from 4- to 6-month-old *Bscl*2^+/+^ males (*N*=3) and *Bscl2*^−/−^ males (*N*=4) using rabbit anti-cleaved caspase-3 (Asp175) antibody (1:300 dilution, Cell Signaling Technology, Danvers, MA, USA) in paraffin sections. Two types of negative control were used: sections from 3-month-old *Bscl2*^−/−^ males incubated with anti-seipin antibody, and sections from 3-month-old *Bscl*2^+/+^ males incubated with normal rabbit IgG (1 : 1000, Santa Cruz Biotechnology, Dallas, TX, USA). All other procedures were the same as described above. Testes from at least three different mice were used.

### *In situ* end-labeling plus

ISEL^+^ detects DNA breaks.^20, 21^ One testis each from 3–4-month-old *Bscl*2^+/+^ and *Bscl2*^−/−^ males (*N*=5) was analyzed. Five consecutive testis sections (10 *μ*m) separated by 200 *μ*m each from each testis were processed for ISEL^+^ labeling as previously described.^20, 21^ All 50 sections (10 mice, 5 sections each) were processed at the same run and low magnification ( × 4) images were taken at the same setting. Only the entire area covered with testis section from each image was quantified using ImageJ (National Institutes of Health, Bethesda, MD, USA) to determine the stained area. ISEL^+^ labeling was expressed as ISEL^+^ labeled area × 100/total area analyzed. The average of all five sections was used to represent each sample for statistical analysis. Testis sections from another set of 3- to 4-month-old mice (*N*=3) were processed for ISEL^+^ labeling and counterstained with DAPI as previously described.^21^

### Annexin V staining

Testes from 5-month-old control (*N*=3) and *Bscl*2^−/−^ (*N*=4) males were decapsulated and the seminiferous tubules were incubated in 0.25% trypsin at 37 °C for 10 min. The trypsin solution was removed and a small piece of the seminiferous tubules was minced in annexin-V binding buffer and subsequently incubated with annexin V (Annexin V-FITC Apoptosis Kit, BioVision, Milpitas, CA, USA) at room temperature for 5 min in the dark. The cells were counterstained with DAPI. At least 100 round spermatids from each male were examined. The percentage of annexin V-positive round spermatids was calculated.

### Mitotracker staining

One cauda epididymis from each of the control mice (*N*=3) and two cauda epididymides from each of the *Bscl*2^−/−^ mice (*N*=4) used for annexin V staining above were collected for staining the mitochondria of sperm. Each sample was minced in 1 ml (for control) or 0.2 ml (for *Bscl*2^−/−^) 1 × PBS in a 1.5-ml microcentrifuge tube and shaken at 37 °C/5% CO_2_ for 5 min. Therefore, the *Bscl*2^−/−^ samples were 10 × concentrated in order to get sufficient sperm density due to low sperm count in the *Bscl*2^−/−^ male. Mitotracker stock solution (MitoTracker Deep Red FM, Life Technologies) was added into the sperm suspensions to a final concentration of 10 *μ*M and incubated in the dark at 37 °C for 10 min. One drop of sperm suspension from each sample was added on a slide, air dried, and counterstained with DAPI.

### Immunofluorescence

Frozen testis sections (10 *μ*m) from 3- to 4-month-old *Bscl*2^+/+^ and *Bscl*2^−/−^ males (*N*=4) were used for detecting the expression of vimentin, PRM1, and PRM2 following the procedure as described previously.^49^ Sections were incubated with vimentin antibody (1:50 dilution, sc-373717, Santa Cruz Biotechnology) or PRM1 antibody (1:100 dilution, 21 *μ*g/ml, Hup1N, Briar Patch Biosciences, Livermore, CA, USA), or PRM2 antibody (1 : 100 dilution, 21 *μ*g/ml, Hup2B, Briar Patch Biosciences) overnight at 4 °C. Vimentin, PRM1, and PRM2 signals were detected by incubating with Alexa Fluor 568 goat anti-mouse antibody (1 : 200 dilution, Life Technologies) for 30–60 min at room temperature. Testis sections were counterstained and mounted in DAPI-containing Vectashield (Vector Laboratories). Two types of negative control were used: sections of 3-month-old *Bscl*2^+/+^ males incubated with mouse IgG (1 : 100, Santa Cruz Biotechnology) or without any primary antibody. All other procedures were the same as described above. In addition, PRM1 and PRM2 were also detected in the sperm spreads from *Bscl2*^+/+^ and *Bscl2*^−/−^ males (4 months old, *N*=3) using the same condition as for testis sections.

### Acrosome labeling

Acrosomes were highlighted with Alexa Fluor 488-conjugated PNA.^52^ Briefly, fixed frozen testis sections from three young adult male mice in each group were treated with 0.3% Triton for 10 min at room temperature. Subsequently, they were incubated with 10 *μ*g/ml PNA in 1% BSA in 1 × PBS for 1 h at room temperature, counterstained with DAPI, and mounted.

### Spermatogenic surface preparations for chromatin analysis

Testes from *Bscl2*^+/+^ and *Bscl2*^−/−^ males (4 months old, *N*=3) were dissected and the tunica albuginea was removed to expose individual seminiferous tubules in 1 × PBS. The seminiferous tubules were immediately processed for cytological analysis of marker proteins of meiotic chromosome synapsis as described previously.^53^ Hypotonic treatment was used to aid in the dissociation of germ cells from the seminiferous tubules and to facilitate meiotic chromosome spreading and subsequent marker protein analysis by chromatin decondensation. Briefly, seminiferous tubules were incubated in a sodium citrate solution (30 mM Tris, 50 mM sucrose, 17 mM trisodium citrate, 5 mM EDTA pH 8.2) for 25 to 35 min at room temperature. Following dissociation, drops of cell suspension were applied to wet glass slides containing 2% paraformaldehyde (Electron Microscopy Services, Hatfield, PA, USA) and 0.15% Triton X-100 (Bio-Rad) in H_2_O, to facilitate nuclear protein cross-linking. Slides were allowed to air dry and then stored at −80 °C until immunochemical analysis.

### Immunofluorescence of spermatogenic surface spreads

Meiotic prophase I staging and the degree of chromosome synapsis in *Bscl2*^+/+^ and *Bscl2*^−/−^ spermatocytes were determined by co-immunochemical detection of the SYCP1 and SYCP3 using polyclonal mouse anti-SYCP3 (Abcam, Cambridge, MA, USA) and polyclonal rabbit anti-SYCP1 (Abcam) antibodies at a 1 : 500 dilution in dilution buffer (1 mg/ml BSA (Sigma) in 1 × PBS, 0.01% Triton X-100). Following overnight incubation at 4 °C and repeated wash steps in dilution buffer, an Alexa Fluor 555 goat anti-mouse (Life Technologies) and an Alexa Fluor 488 goat anti-rabbit secondary antibody were applied at a dilution of 1 : 1000 for 1 h at room temperature. The male germ cells were then counterstained and mounted in DAPI-containing Vectashield (Vector Laboratories). Similarly, the subnuclear localization of pericentric heterochromatin domains was detected using a rabbit anti-H3K9me3 antibody (1 : 400, Upstate, Charlottesville, VA, USA). Immunofluorescence and chromatin configurations were visualized using a Leica DMRE fluorescence microscope (Buffalo Grove, IL, USA), and images were captured using a Leica DFC 350 F CCD camera. At least 100 randomly selected round spermatids from each sample were examined for the presence of chromocenter fragmentation. At least 100 randomly selected elongating spermatids and sperm from each sample were examined for the presence of chromatin vacuoles. The percentages of round spermatids with chromocenter fragmentation, as well as elongating spermatids and sperm with chromatin vacuoles, were quantified.

### Statistical analyses

Data are presented as mean±S.D. Wilcoxon rank sum test was used for plugging latency. The *χ*^2^-test was used for pregnancy rate and plugging rate. Two-tailed unequal variance Student's *t*-test was used for the rest of the parameters. For the parameters with percentages, Student's *t*-test was performed after arcsine transformation. The significance level was set at *P*<0.05.

## Abbreviations

ATR: Rad3-related protein
*Bscl2*, seipin: Berardinelli–Seip congenital lipodystrophy type 2
DAPI: 4',6-diamino-2-phenylindole
ER: endoplasmic reticulum
H3K9me3: histone H3 trimethylated at lysine 9
HRP: horseradish peroxidase
ISEL^+^: *in situ* end-labeling plus
mES: mouse embryonic stem
PA-PLA_1_: phosphatidic acid-preferring phospholipase A_1_
PNA: peanut agglutinin
PND: postnatal day
PRM1/2: protamines 1 and 2
SERCA: sarco/ER Ca^2+^-ATPase
SSC: saline-sodium citrate
SYCP1/3: synaptonemal complex proteins 1 and 3
