# Supplementary material for: Seipin deficiency increases chromocenter fragmentation and disrupts acrosome formation leading to male infertility
Source: Cell Death Dis. 2015 Jul 16;6(7):e1817–. doi: 10.1038/cddis.2015.188 (PMC4650735; doi:10.1038/cddis.2015.188)
Supplement: Supplementary Information [file cddis2015188x1.doc]

**Supplementary Figure Legends**

**Figure S1.** Male fertility test, sperm analyses, and quantification of seminiferous tubules. +/+, *Bscl2*^+/+^; +/-, *Bscl2*^+/-^; -/-, *Bscl2*^-/-^. A-E: data from males at the end of 2 months fertility test. A. Relative testis weight. B. Epididymis weight. C. Relative epididymis weight. D. Weight of seminal vesicle and coagulating gland. E. Relative weight of seminal vesicle and coagulating gland. A-E: N=7 (+/+) and 10 (-/-). F. Sperm count for determining percentage of motile sperm. N=6 (+/+ & +/-) and 9 (-/-). G. Sperm count in the cauda epididymis of mated males 1 week after mating. H. Sperm count in the uterus of the mated females in the morning after mating. G-H: N=5 (+/+) and 7 (-/-). I. Number of seminiferous tubules in 20x testis histology images. J. Number of stages IX-XI seminiferous tubules in 20x testis histology images. I-J: N=6 (+/+) and 5 (-/-), 3-6 months old. A-J: error bar, standard deviation; *, P<0.05.

**Figure S2.** Expression of *Bscl2* mRNA in *Bscl2*^+/+^ testis, coagulating gland, and seminal vesicle. A. Upregulation of *Bscl2* mRNA in testis from postnatal day 15 (PND15) to 3 months (3M) old by quantitative PCR. N=5 (PND15) and 3 (3M); error bar, standard deviation; *, P=0.069. B-F: in situ hybridization; tissues from 3 months old males. B. Localization of *Bscl2* mRNA in epithelium of a coagulating gland. Antisense (AS) probe; scale bar, 500 µm. C. Enlarged image of the rectangle area in B. Scale bar, 50 µm. D. Localization of *Bscl2* mRNA in epithelium of a seminal vesicle. Antisense probe; scale bar, 200 µm. E. Enlarged image of the rectangle area in D. Scale bar, 50 µm. F. A seminal vesicle section incubated with a sense probe as the negative control.

Figure S3. Detection of cleaved caspase-3 using immunohistochemistry in 4-6 months old *Bscl2*^+/+^ (+/+) and *Bscl2*^-/-^ (-/-) testes. A-C. *Bscl2*^+/+^ testis. D-F. *Bscl2*^-/-^ testis. Red arrows, cleaved caspase-3 positive germ cells; scale bars, 200 μm (A, D), or 50 μm (B, E), or 32 μm (C, F).

**Figure S4.** In situ end-labeling plus (ISEL^+^) of 3-4 months old *Bscl2*^+/+^ (+/+) and *Bscl2*^-/-^ (-/-) testes. One testis from each mouse (N=5 per group) was analyzed. Mouse number from each group was indicated on the left. Five consecutive testis sections (10 μm) separated by 200 µm each from each testis were shown. All these sections were quantified and the average of the five sections from each mouse was used for quantification shown in Figure 4A. A. *Bscl2*^+/+^ testis sections. B. Enlarged image of the rectangle area in the third section of male No. 3. C. Enlarged image of the rectangle area in B. D. *Bscl2*^-/-^ testis sections. E. Enlarged image of the rectangle area in the third section of male No. 8. F. Enlarged image of the rectangle area in E. Red arrows indicating ISLE^+^ labeled spermatids. C & F: Green arrows indicating clusters of ISLE^+^ labeled germ cells. Scale bars: 500 µm in A & D; 200 µm in B & E; and 50 µm in C & F.

**Figure S5.** Homologous chromosome synapsis in spermatocytes. A. Blue DAPI staining of a *Bscl2*^+/+^ (+/+) spermatocyte. B. Red SYCP3 staining of the spermatocyte in A. C. Green SYCP1 staining of the spermatocyte in A. D. Merged image of B and C. E. Merged image of A, B, and C. F. Blue DAPI staining of a *Bscl2*^-/-^ (-/-) spermatocyte. G. Red SYCP3 staining of the spermatocyte in F. H. Green SYCP1 staining of the spermatocyte in F. I. Merged image of G and H. J. Merged image of F, G, and H. Arrows, autosomes; XY, position of the sex chromosomes at the XY body. Scale bar, 10 µm.

**Figure S6.** Expression of protamine 1 and 2 mRNAs and proteins in testis. Males at 3-4 months old were used in the studies. A-C. mRNA expression of protamine 1 (*Prm1*) (A), *Prm2* (B), and hypoxanthine phosphoribosyltransferase 1 (*Hprt1*) (C) in *Bscl2*^+/+^ (+/+) and *Bscl2*^-/-^ (-/-) testis. N=5; error bars, standard deviation; * P<0.05. D. DAPI staining of *Bscl2*^+/+^ seminiferous epithelium. E. Protamine 1 staining of the section in D. F. Merged image of D and E. G. DAPI staining of *Bscl2*^-/-^ seminiferous epithelium. H. Protamine 1 staining of the section in G. I. Merged image of G and H. J. DAPI staining of *Bscl2*^-/-^ sperm spread. A round spermatid on the left side and a late spermatid with chromatin vacuoles on the right side. K. Protamine 1 staining of the late spermatid in J. L. Merged image of J and K. M. DAPI staining of *Bscl2*^+/+^ seminiferous epithelium. N. Protamine 2 staining of the section in M. O. Merged image of M and N. P. DAPI staining of *Bscl2*^-/-^ seminiferous epithelium. Q. Protamine 2 staining of the section in P. R. Merged image of P and Q. Scale bar, 100 µm in D-I and M-R, 10 µm in J-L.
