# Supplementary figures and images for: Seipin deficiency increases chromocenter fragmentation and disrupts acrosome formation leading to male infertility
Source: Cell Death Dis. 2015 Jul 16;6(7):e1817–. doi: 10.1038/cddis.2015.188 (PMC4650735; doi:10.1038/cddis.2015.188)

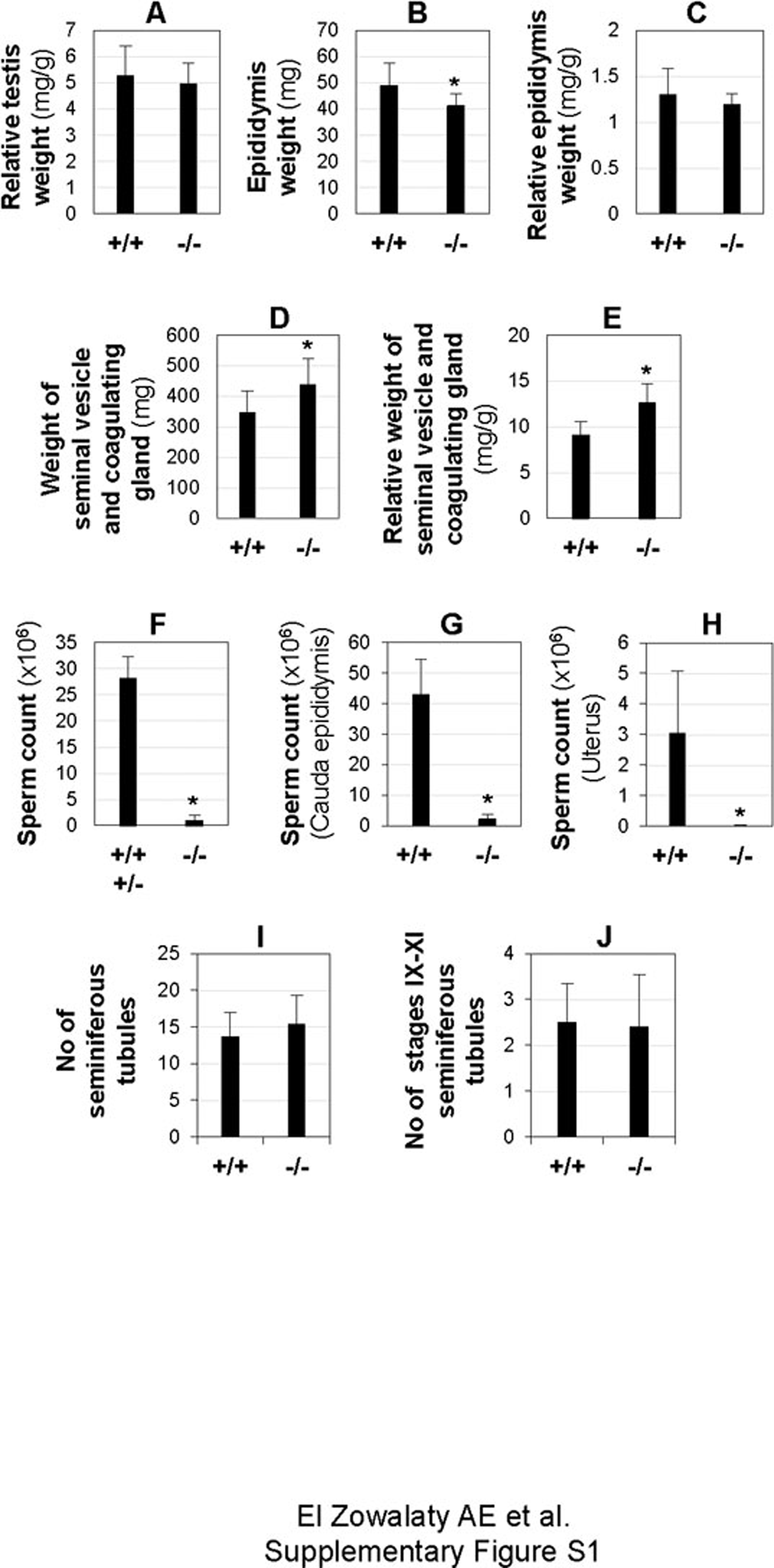

Supplement: Supplementary Figure 1 [file cddis2015188x2.tif]

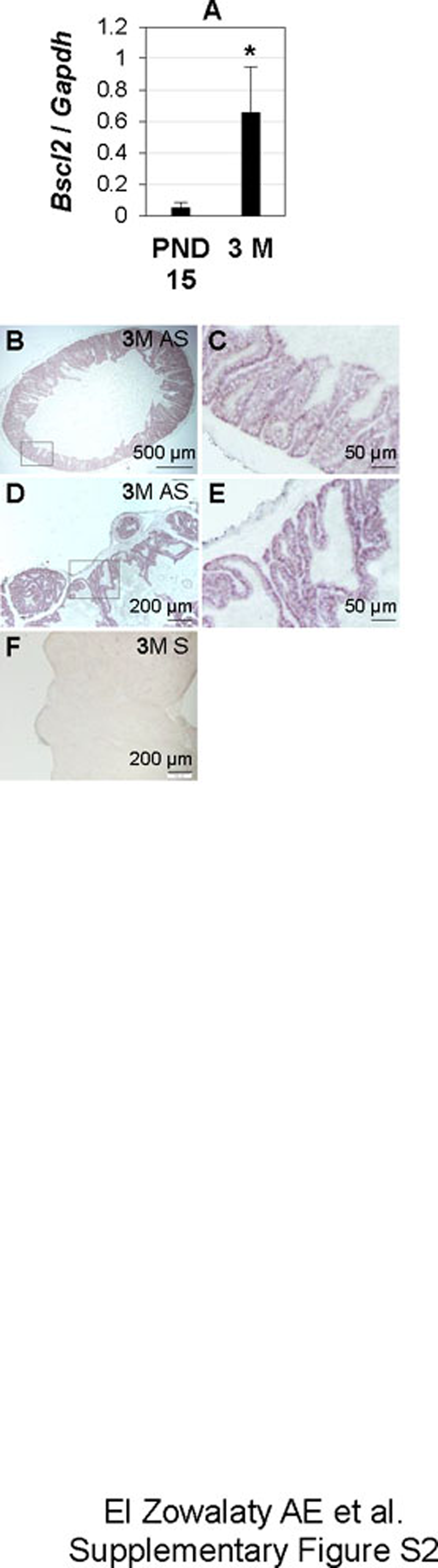

Supplement: Supplementary Figure 2 [file cddis2015188x3.tif]

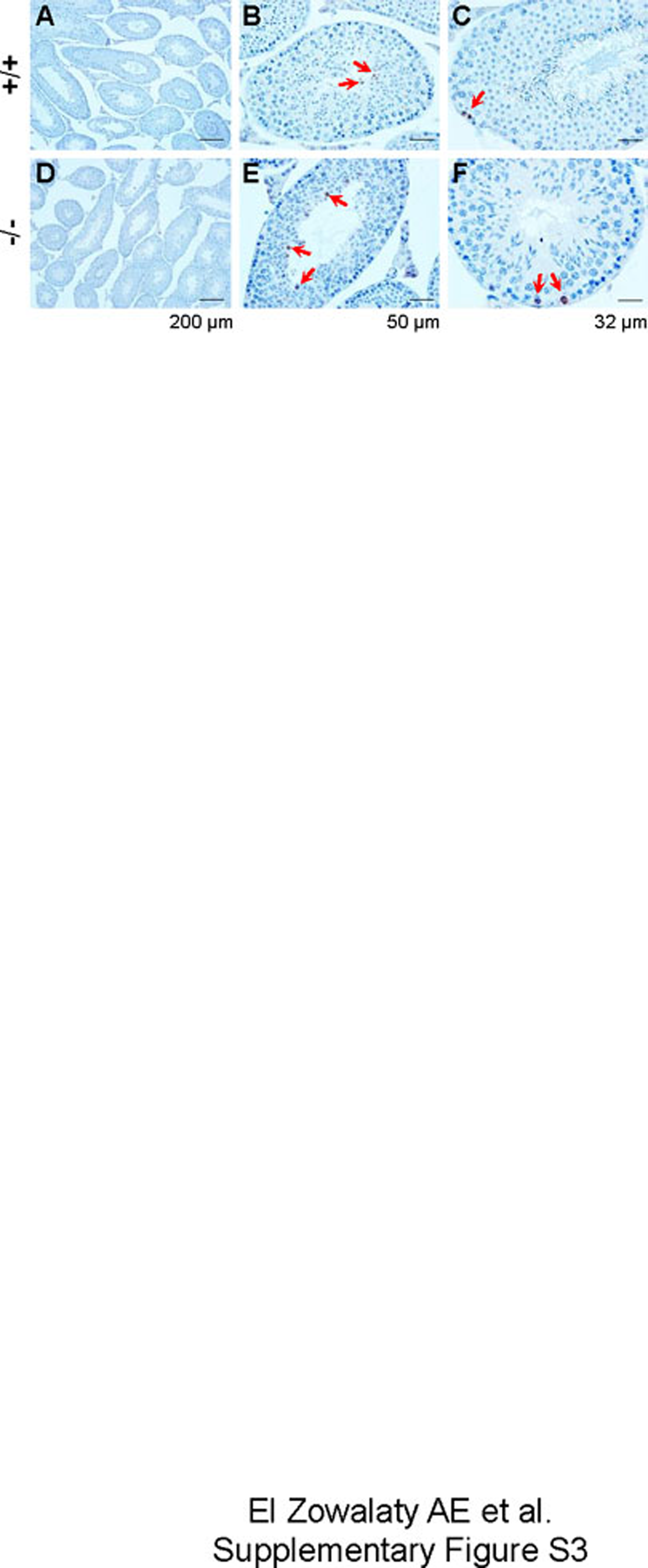

Supplement: Supplementary Figure 3 [file cddis2015188x4.tif]

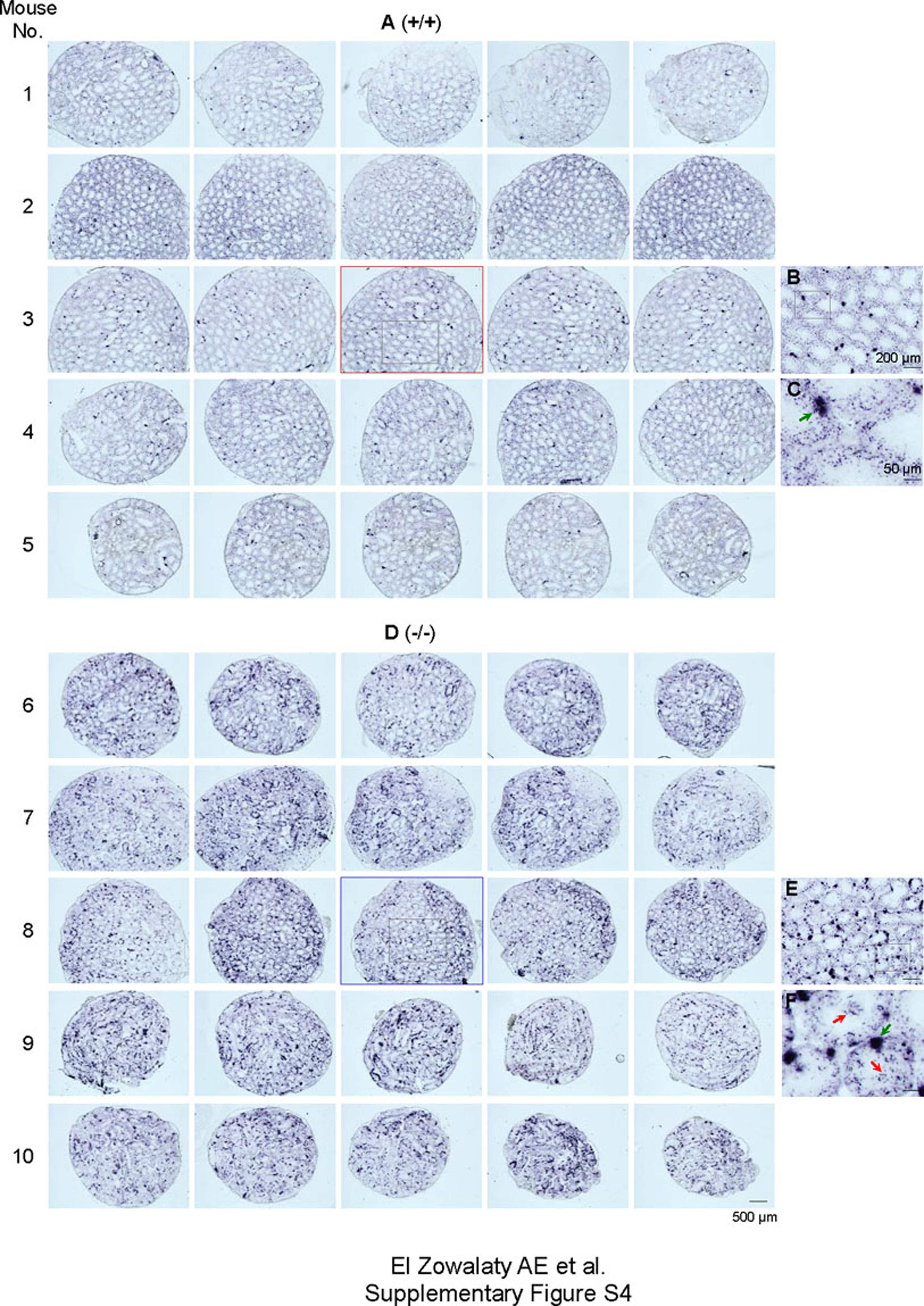

Supplement: Supplementary Figure 4 [file cddis2015188x5.tif]

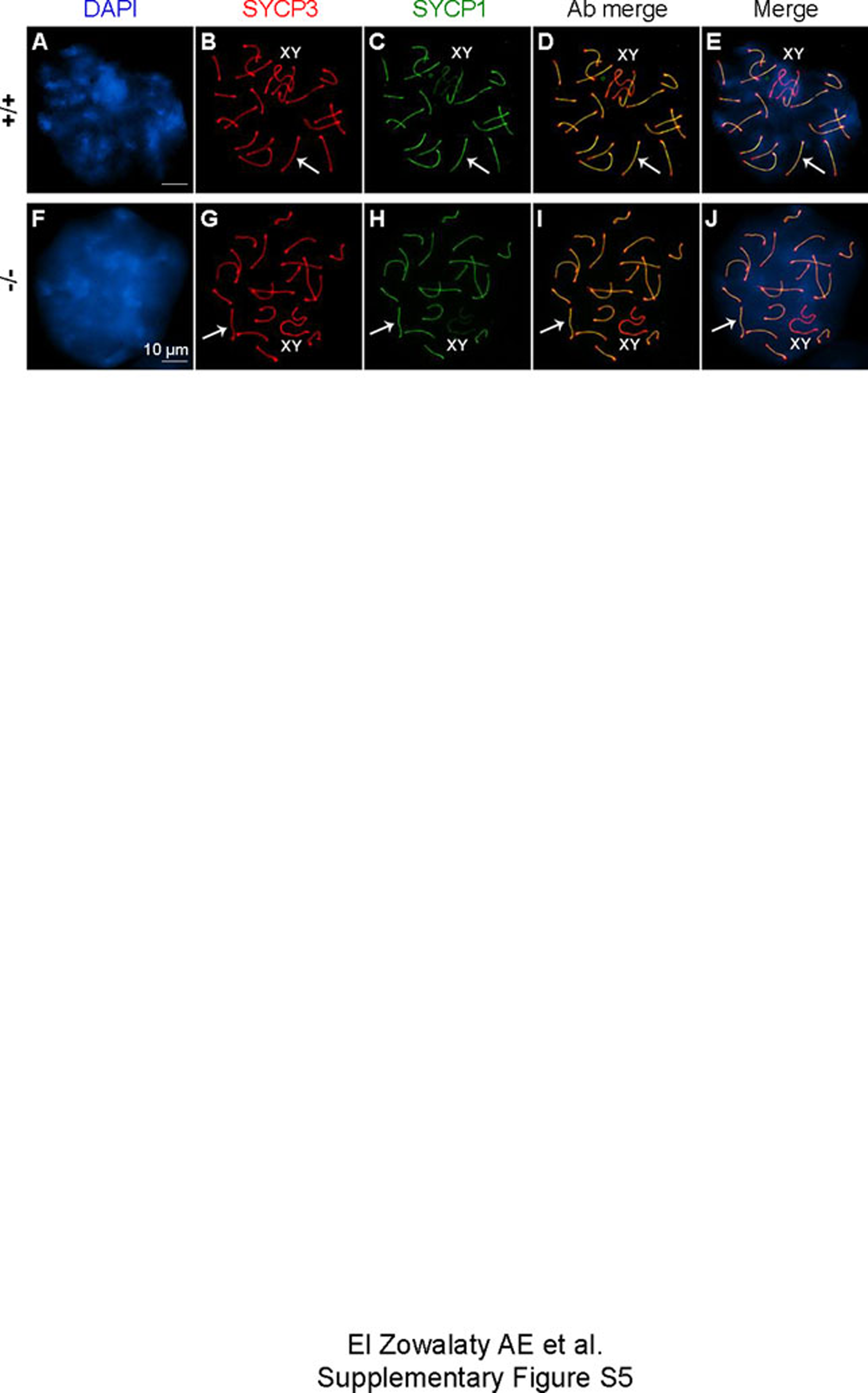

Supplement: Supplementary Figure 5 [file cddis2015188x6.tif]

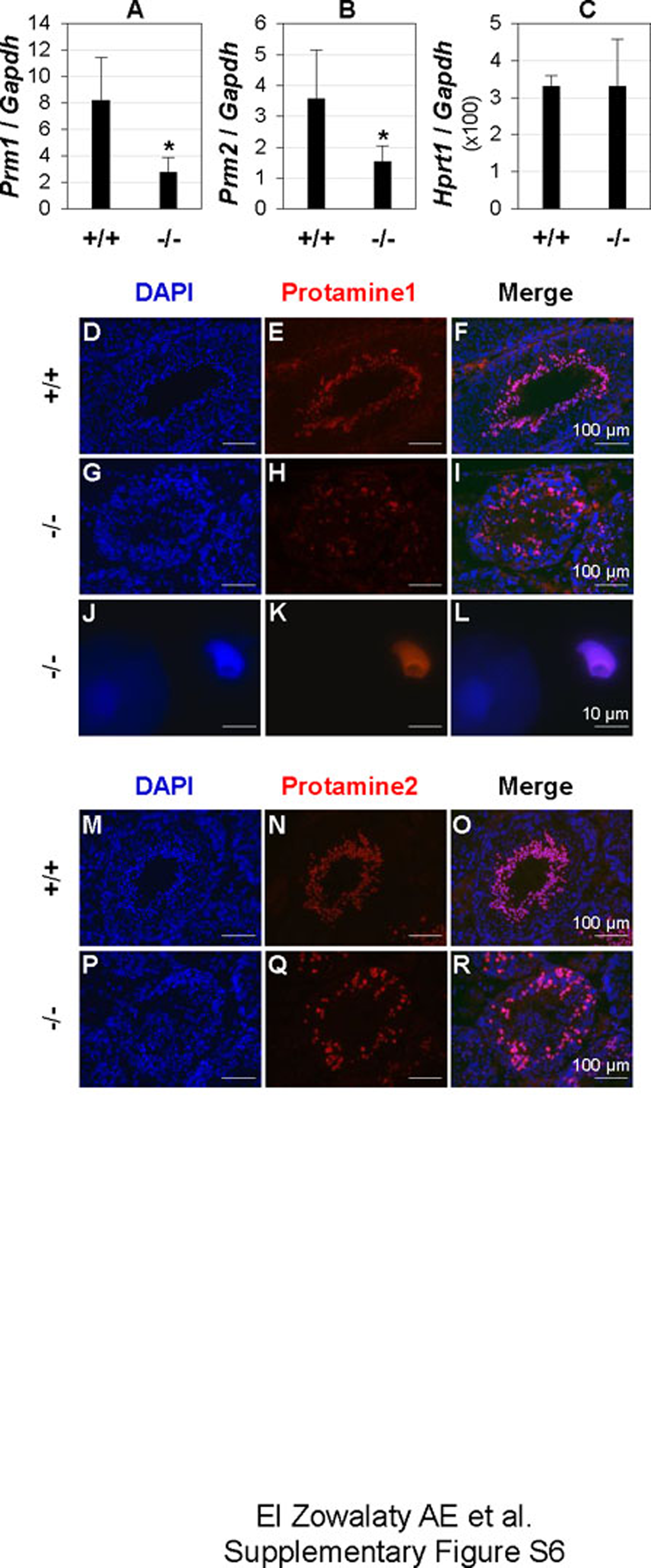

Supplement: Supplementary Figure 6 [file cddis2015188x7.tif]
